# Periacetabular Osteotomy with a Modified Fixation Technique Using K-Wires Shows Clinical Results Comparable to Screw Fixation at Mid-Term Follow-Up

**DOI:** 10.3390/jcm12196204

**Published:** 2023-09-26

**Authors:** Vincent Justus Leopold, Christian Hipfl, Robert Karl Zahn, Matthias Pumberger, Carsten Perka, Sebastian Hardt

**Affiliations:** Center for Musculoskeletal Surgery, Charité-University Medicine Berlin, Charitéplatz 1, 10117 Berlin, Germany

**Keywords:** developmental dysplasia of the hip, periacetabular osteotomy, surgical technique, fixation options, patient outcomes

## Abstract

Background: The optimal fixation technique in periacetabular osteotomy (PAO) remains controversial. Modified fixation with Kirschner wires (K-wires) was described as a feasible and safe alternative. However, clinical follow-up of patients treated with this technique is lacking. Aims: To assess patient-reported outcomes (PROMs) in patients treated with PAO with the K-wire fixation technique and to compare it with the screw fixation technique. Methods: We conducted an analysis of 202 consecutive PAOs at a single university center between January 2015 and June 2017. A total of 120 cases with complete datasets were included in the final analysis. PAOs with K-wire fixation (*n* = 63) were compared with screw fixation (*n* = 57). Mean follow-up was 63 ± 10 months. PROMs assessed included the International Hip Outcome Tool (iHOT 12), Subjective Hip Value (SHV), and UCLA activity score (UCLA). Pain and patient satisfaction (NRS) were evaluated. Joint preservation was defined as non-conversion to total hip arthroplasty (THA). Results: Preoperative baseline PROMs in both fixation groups were similar. In both groups, PROMs (*p* = <0.001) and pain (*p* = <0.001) improved significantly. Postoperative functional outcome was similar in both groups: iHOT 12 (71.8 ± 25.1 vs. 73 ± 21.1; *p* = 0.789), SHV (77.9 ± 21.2 vs. 82.4 ± 13.1; *p* = 0.192), UCLA (6.9 ± 1.6 vs. 6.9 ± 1.9; *p* = 0.909), and pain (2.4 ± 2.1 vs. 2.0 ± 2.1; *p* = 0.302). Patient satisfaction did not differ significantly (7.6 ± 2.6 vs. 8.2 ± 2.2; *p* = 0.170). Conversion to THA was low in both groups (two vs. none; *p* = 0.497). Conclusion: Periacetabular osteotomy with K-wire fixation provided good clinical results at mid-term follow-up, comparable to those of screw fixation. The technique can therefore be considered a viable option when deciding on the fixation technique in PAO.

## 1. Introduction

Periacetabular osteotomy (PAO) is the standard for the surgical treatment of developmental dysplasia of the hip (DDH), with the goal of improving the acetabular coverage of the femoral head and preserving the native hip joint [1]. At the time of surgery, patients are usually young and have high functional demands. Patients who undergo PAO seek to improve joint function and preserve their native hip joint. Through defined osteotomies the acetabulum is liberated from the pelvis and after three-dimensional reorientation is fixed in place, thus improving femoral head coverage. Since the first description of the technique by Ganz et al. [1,2], different fixation techniques of the acetabular fragment have been described [3,4,5]. Predominantly, towards the end of the procedure after acetabular reorientation, the intermediate fixation with K-wires is dissolved and a definitive fixation is achieved with screws [1,6]. For this original technique, good clinical results were reported in both mid-term and long-term follow-up [7,8,9]. As an alternative, a fixation technique with definitive fixation using K-wires has been described and a fixation stability equivalent to that of screw fixation was observed [10,11]. However, data on clinical outcomes and joint preservation in patients treated with this fixation technique in PAO are lacking to date.

The aim of the present study was therefore to evaluate the clinical outcome and joint survival of patients undergoing PAO with K-wire fixation and to compare it with that of patients with PAO and classic screw fixation at mid-term follow-up [12]. The null hypothesis was that the modified technique with K-wire fixation would not have inferior outcomes compared to the classic technique using screws.

## 2. Methods

We performed a retrospective analysis of data from patients who had received PAO for DDH at a single university center between January 2015 and June 2017. The prior approval of the local ethics committee was obtained (EA1/052/21).

**Figure 1:**
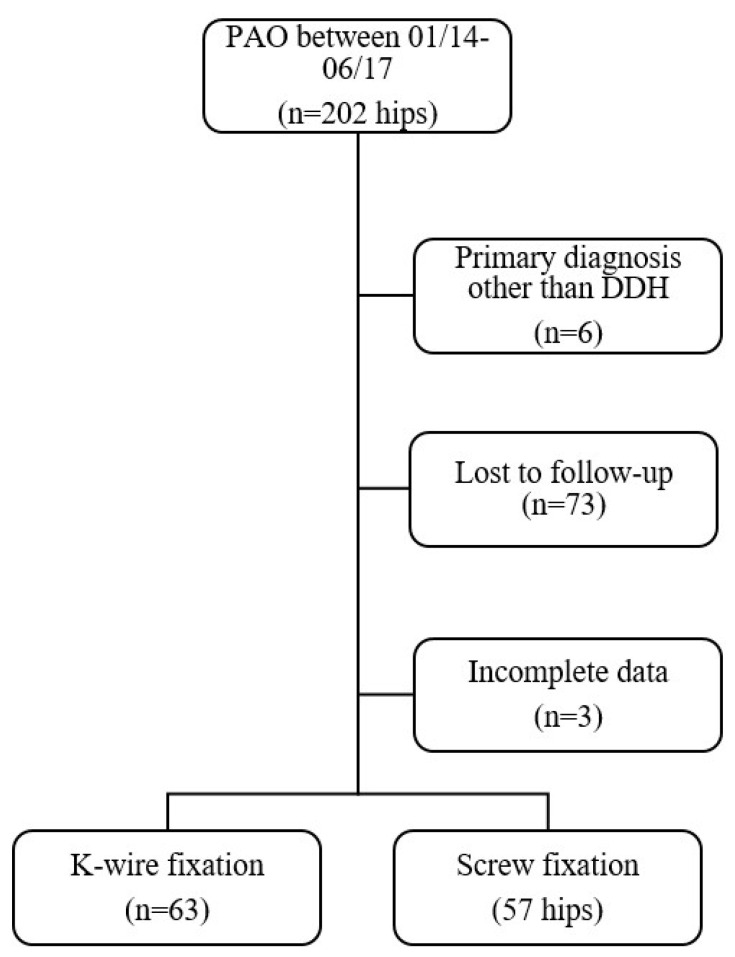
CONSORT Flowchart illustrating study population and reasons for exclusion.

While the present study describes a unique analysis, some patient data may have been reported in other studies. The study was conducted in accordance with the STROBE guidelines. A total of 202 consecutive PAOs in 173 patients were performed during this period. Inclusion criteria were patients with a primary diagnosis of DDH treated with PAO with completed questionnaires including clinical data (see below), as well as informed consent. Exclusion criteria were a primary diagnosis other than DDH (i.e., acetabular retroversion), prior operation on the ipsilateral hip joint, or incomplete data at follow-up. Only complete datasets were included in the final analysis. Patient age was not an exclusion criterion. However, according to previously published data in the literature, the indication for PAO was generally only up to 40 years of age. Beyond that, only patients with biologically younger age and joint status became candidates for PAO [13,14,15]. To be able to report a mid-term follow-up according to the definition in the literature, the inclusion period was chosen accordingly [12]. For a detailed overview of patient selection, see Figure 1.

### 2.1. Surgical Technique

**Figure 2:**
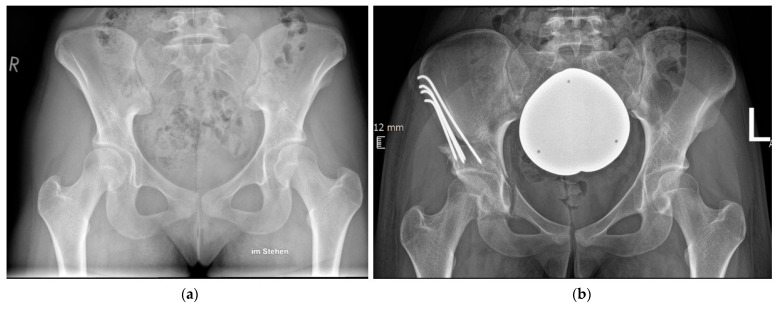
(**a**) Preoperative a.p. radiograph before PAO. (**b**) Postoperative radiograph after PAO and fixation with K-wires.

The surgical procedure of PAO has been previously described [1,13]. In the studied cohort, acetabular reorientation and fixation was achieved under fluoroscopic guidance using an ilioinguinal approach [1]. Fixation of the acetabular fragment was achieved either through four to five unthreaded K-wires (2.5 mm) bent, cut, and recessed into the iliac crest or three to four fully threaded screws (4.5 mm). The target of intraoperative reorientation was defined as a lateral center edge angle (LCEA) between 30° and 35°, acetabular index (AI) between 0° and 5°, and femoral head extrusion index (FHEI) between 17% and 26%, with an anteverted acetabulum without cross-over sign. All PAOs were performed by four senior hip surgeons trained in periacetabular osteotomy and well beyond their learning curves. The choice of fixation technique was based on the surgeon’s preference and regardless of the extent of dysplasia. Surgeons used either K-wire or screw fixation. None used both techniques. Figure 2 shows the case of a dysplastic hip treated with PAO using the described modified technique with definitive K-wire fixation.

### 2.2. Postoperative Mobilization

Patients in both groups were mobilized in the same way using a standard mobilization regimen allowing tip-touch partial weight-bearing of the operated limb for the first 6 weeks postoperatively. Weight-bearing was then increased to half of the patient’s body weight till the 10th postoperative week, with a gradual increase to full weight-bearing thereafter until 3 months postoperatively [11].

### 2.3. Radiological Assessment

All patients received standardized standing a.p. pelvis radiographs. Radiological parameters relevant for DDH were measured preoperatively, postoperatively after initial mobilization, and at 3 months follow-up. Lateral center edge angle (LCEA), Tönnis angle (TA), and femoral head extrusion index (FHEI) were measured. Grade of osteoarthritis was graded according to Tönnis. All patients had a grade of osteoarthrosis of ≤1 according to Tönnis and showed at least one radiologic abnormality, including a lateral center edge angle of Wiberg (LCE) less than 25° [16,17], an acetabular inclination (AI) greater than 10° [16], an anterior center edge angle (ACEA) as described by Lequesne and de Seze of less than 25° [18], and a femoral head extrusion index (FHEI) as described by Heyman and Herndeon of greater than 26% [19]. Femoral head congruency was preoperatively determined by 30° abduction functional radiographs and was good in all hips. Radiographs were furthermore evaluated for loss of correction. A loss of correction was defined as the difference between initial correction at immediate postoperative time and at 3 months follow-up. A clinically significant loss of correction was defined as a loss of the acetabular fixation requiring revision surgery or a delta LCEA of more than 5° as measured in the radiological assessment. All measurements were performed by two residents both trained by the same senior orthopedic surgeon. For a detailed overview of the demographic and radiological data, see Table 1. 

### 2.4. Clinical Assessment

Clinical outcome assessment included hip joint specific function, hip joint preservation and patient satisfaction. Pre- and postoperative hip function was assessed by means of a standardized set of patient-reported outcome measures (PROMs). Data collected included pain level using the Numerical Rating Scale (NRS), International Hip Outcome Tool (iHOT 12), Subjective Hip Value (SHV), and UCLA activity score (UCLA) [20,21,22,23]. Failed hip joint preservation was defined as conversion to total hip arthroplasty. Patient satisfaction was assessed using a numerical scale ranging from 1 to 10. Clinical data were collected at follow-up via a hip-specific questionnaire.

Any subsequent surgeries with conversion to total hip arthroplasty (THA) or revision surgery were also recorded. Furthermore, implant-associated complications were recorded, including soft tissue irritation requiring implant extraction surgery. There was no routine protocol for implant removal and implant extraction surgery was only performed in cases of symptomatic implant-associated soft tissue irritation, with radiologically healed osteotomies and not earlier than one year postoperatively. Implant migration was defined as the migration of the used implant by more than 5 mm. Implant failure was defined as the breaking of the implant. A clinically significant loss of correction was defined as a loss of the acetabular fixation requiring revision surgery or a delta LCEA of more than 5° as measured in the radiological assessment. Duration of surgery was defined as cut–suture time and was also recorded.

### 2.5. Statistical Analysis

Frequency rates, means and range were utilized to describe baseline patient characteristics. The normal distribution was tested using the Shapiro–Wilk test. A *t*-test was used to determine significant differences between continuous data, and Fisher’s exact test for categorical data. A *p*-value of less than 0.05 was considered statistically significant. For documentation of the collected data, Microsoft Excel (Microsoft Corp. Released 2016. Excel Professional for windows, Version 16.16.2. Redmond, WA, USA: Microsoft Corp.) was used. The collected data were analyzed using IBM SPSS 25 (IBM Corp. Released 2017. IBM SPSS Statistics for Windows, Version 25.0. Armonk, NY, USA: IBM Corp.).

## 3. Results

Out of the initial 202 hips, 6 were operated on for indications other than DDH. In 73 cases, the patient could not be invited for follow-up assessment due to a change of address or telephone number and was therefore lost to follow-up. These missing data were considered as missing at completely random (MCAR). Three patients did not provide complete data; thus, 120 hips with complete datasets were included in the final analysis. Of these hips, 63 underwent definitive fixation using K-wires (K-wire group). Fixation with screws served as a control group, with 57 hips (Screw group). Medium follow-up was at 63 ± 10 months. Mean age at surgery (*p* = 0.206), mean BMI (*p* = 0.994), and gender distribution (*p* = 0.306) did not differ significantly between both groups. With regard to radiological parameters, the K-wire group showed slightly more dysplastic values for LCEA (*p* = 0.039) and TA (*p* = 0.008). Both groups were corrected to a comparable extent with no significant differences observed for correction values of radiological parameters. For a detailed overview of radiological data, see Table 1.

In both groups, pain as measured by NRS (*p* = <0.001) and function as measured by the above-mentioned PROMs (*p* = <0.001) improved significantly. Preoperative values for pain on NRS did not differ significantly between both groups preoperatively (*p* = 0.744) as well as postoperatively (*p* = 0.302). Preoperative joint function as measured using the iHOT-12 (*p* = 0.872) and SHV (*p* = 0.915) did not differ significantly between both groups. Activity level as measured using the UCLA was not significantly different in both groups preoperatively (*p* = 0.073). At follow-up, neither pain (*p* = 0.302) nor joint function as measured by iHOT-12 (*p* = 0.789) and SHV (*p* = 0.192) differed significantly between both groups. The UCLA activity score was also not significantly different between both groups at follow-up (*p* = 0.909). Patient satisfaction was also similar for both groups as no significant difference was observed (*p* = 0.170). For a detailed summary of the comparison of pre- and postoperative PROMs, see Table 2. At follow-up, a total of two patients had been converted to total hip arthroplasty (THA) in group 1 compared with none in group 2. No significant difference was observed between the two groups (*p* = 0.497). In the K-wire group, one patient received hip arthroscopy for femoroacetabular impingement and one patient received revision PAO for overcorrection, which was not due to fragment dislocation. In the Screw group, one patient received hip arthroscopy. Concerning implant-associated complications, there was a significantly larger proportion of patients with soft tissue irritation necessitating implant removal surgery in the K-wire group (*p* = 0.0001). No significant difference was observed for implant migration (*p* = 0.095). In both groups, there were no cases of implant failure, intra-articular implant migration or a clinically significant loss of correction. For a detailed overview of implant-associated complications, see Table 3.

## 4. Discussion

While numerous clinical results of PAO with the classical screw fixation technique have been reported [24,25,26], detailed clinical results for the technique with K-wire fixation are lacking so far. The most important finding of the work presented is that both pain and joint-specific function improved significantly in patients undergoing PAO with the K-wire technique. Compared with the previously established screw fixation technique, there was a similar improvement in pain and PROMs with no significant differences being observed between both techniques. The conversion rate to THA as well as revision surgery was low in both groups and did not differ significantly. Subsequent implant extraction surgery due to soft tissue irritation was performed significantly more often if K-wires were used. Patient satisfaction was also comparable in both groups.

Since the introduction of the PAO technique, the central endpoint of the procedure has been survival of the native hip joint [1,8]. Predictive factors for joint preservation were defined by the analysis of long-term outcomes, such as age, degree of osteoarthritis, and preoperative function [8,14]. However, the prerequisite for the survival of the hip joint is the improvement of the biomechanics of the joint to values as close to physiological as possible, which can be achieved by PAO [27,28]. It is crucial not only to establish these physiological biomechanics but also to maintain them through adequate fixation until bony consolidation, as loss of correction might result in PAO failure [4,11,27]. For the fixation technique under investigation, it was previously shown that given an appropriate mobilization regimen, it provides sufficient stability for this purpose, comparable to established screw fixation [11]. Regarding the important endpoint of hip joint preservation, the results of our study show equivalent rates of joint preservation at mid-term follow-up with both the fixation technique with K-wires and with screws, as the conversion rate to THA was low and did not differ significantly between both groups. Furthermore, implant-associated complications were low in both groups and no clinically significant loss of correction occurred.

However, since patients undergoing PAO are mostly young, often active individuals with high functional demands, postoperative function is also of great importance beyond the survival of the native hip joint [24,26,29]. PROMs specifically suited for follow-up of PAO patients have been previously defined in the literature [23]. By follow-up of PAO patients using these hip-specific PROMs, it has been shown that joint-specific function improves significantly after PAO [25]. These studies followed up patients who had received PAO using the classical fixation technique with screws. With the data from our studied cohort, we demonstrated that clinical improvement in hip-joint-specific function with the use of K-wire fixation was equivalent to the original technique using fixation with screws (see Table 2). 

Another important factor in deciding for or against a surgical technique is potential postoperative complications. A previous study comparing the implant-related complications of K-wire fixation and screw fixation in PAO showed a significantly higher rate of soft tissue irritation necessitating implant removal surgery [11], which was again observed in our study cohort. With a higher rate of this complication and subsequent surgery, lower patient satisfaction could be expected. Even though our study demonstrated equivalent patient satisfaction for the technique under investigation at mid-term, the significantly higher frequency of this complication favors the technique with screw fixation in this regard (see Table 2 and Table 3).

With the exception of soft tissue irritation, no significantly different complication rates, such as implant failure, implant migration, or loss of correction, were previously observed for the K-wire technique [11], which was again observed in the studied cohort (see Table 3). 

Possible advantages of K-wire fixation in PAO, on the other hand, are the potentially reduced rate of non-unions as well as the shortened operation time, as shown by our data and previous studies [10,11]. Reduced rates of non-unions are achieved by semirigid fixation, which allows for minimal movement of the fragment and thus improves osseous healing of the osteotomies [10,11]. A shorter operation time can be achieved since no change from temporary fixation to definitive screw fixation is required, thus eliminating one surgical step [11]. As the additional step of changing temporary to definitive fixation also represents a possible source of loss of correction, eliminating this step is desirable. 

Another advantage could be the size of the implants, since the K-wires used have a significantly smaller diameter (2.5 mm) than the regularly used screws (4.5 mm) [1,4,11,13]. This could be especially advantageous in potentially fragile situations with thin pelvic bones, where thinner implants can be inserted more safely and no additional drilling is required.

The current study is subject to several limitations. First is the retrospective study design and lack of randomization of patients. However, at baseline, the two groups were comparable in terms of demographic data and preoperative baseline clinical parameters, showing no significant differences for the measured PROMs. Second, the presented study collective was not a single-surgeon series, because of which reduced operation time cannot be attributed with certainty to the choice of fixation. However, all surgeons were senior orthopedic surgeons well beyond their learning curve. Third, in the present study, groups differed with regard to preoperative radiological parameters and the K-wire group had more dysplastic baseline values. However, correction delta was not significantly different and radiological parameters were improved to physiological parameters postoperatively in both groups, and it was shown that even in the case of more severe dysplasia, fixation with K-wires in PAO is effective and provides sufficient stability. Fourth is the relatively large loss-to-follow-up, since several patients could not be reached for follow-up due to changes in contact information. However, with the data not missing systemically but at random, we do not consider there to be substantial bias as a result of the missing data, and we were still able to follow-up a large number of patients and report for the first time in the literature a clinical follow-up of the studied technique.

## 5. Conclusions

The k-wire fixation technique in PAO shows good clinical results at mid-term follow-up. Improvements in joint-specific function and patient satisfaction are equivalent to those of the previously described screw fixation technique. The results of this study can be considered by orthopedic surgeons when deciding on the fixation technique in PAO. High-quality prospective studies are required before definitive recommendations about the best method of fixation after PAO can be made.

## Figures and Tables

**Table 1:** Patient demographics and radiological parameters pre- and postoperatively. Significant differences are indicated by bold numbers.

	K-Wire Fixation (*n* = 63)	Screw Fixation (*n* = 57)	*p*-Value
Age	28.8 ± 7.3	27 ± 8.1	0.206
BMI	24.2 ± 4.3	24.2 ± 4.8	0.994
Gender w/m	56/7	46/11	0.306
LCEA preOP	15.0 ± 6.5	17.3 ± 5.2	**0.039**
LCEA postOP	28.2 ± 5.7	30.7 ± 5.8	**0.021**
Correction	13.1 ± 6.7	13.6 ± 6.5	0.694
TA preOP	14.8 ± 6.8	11.5 ± 6.0	**0.008**
TA postOP	3.5 ± 7.6	−1.2 ± 6.2	**0.0001**
Correction	−11.2 ± 6.6	−12.5 ± 6.8	0.282
FHEI preOP	25.2 ± 9.4	22 ± 6.1	**0.030**
FHEI postOP	11 ± 9.6	8.1 ± 7.1	0.076
Correction	−14.2 ± 7.1	−13.8 ± 6.2	0.720

**Table 2:** Patient-reported outcomes, pain and patient satisfaction compared between both fixation groups pre- and postoperatively.

	K-Wire Fixation (*n* = 63)	Screw Fixation (*n* = 57)	*p*-Value
NRS pre OP	7.2 ± 2	7.3 ± 1.6	0.744
NRS post OP	2.4 ± 2.1	2.0 ± 2.1	0.302
Difference	4.8 ± 2.5	5.3 ± 2.2	0.250
Patient satisfaction	7.6 ± 2.6	8.2 ± 2.2	0.170
iHOT-12			
Preoperative	41.3 ± 21.6	40.7 ± 22.8	0.872
Postoperative	71.8 ± 25.1	73 ± 21.1	0.789
Difference	30.5 ± 28.7	31.6 ± 28.8	0.832
SHV			
Preoperative	41.9 ± 26.5	42.4 ± 21.6	0.915
Postoperative	77.9 ± 21.2	82.4 ± 13.1	0.192
Difference	35.4 ± 33.2	37.8 ± 25.1	0.658
UCLA			
Preoperative	5.5 ± 2.5	4.6 ± 2.4	0.073
Postoperative	6.9 ± 1.6	6.9 ± 1.9	0.909

**Table 3:** Comparison between K-wire and screw fixation regarding implant-associated complications (number of cases) and surgical time (minutes). Surgical time was defined as cut–suture time. Implant extraction surgery was performed in cases of symptomatic soft tissue irritation. Implant migration was defined as implant migration of more than 5 mm. Implant failure was defined as the breaking of the implant. A clinically significant loss of correction was defined as a loss of the acetabular fixation requiring revision surgery or a delta LCEA of more than 5° as measured in the radiological assessment. Bony consolidation was determined on postoperative a.p. pelvic X-rays. Significant differences are indicated by bold numbers.

	K-Wire Fixation (*n* = 63)	Screw Fixation (*n* = 57)	*p*-Value
Operating time (min)	93.2 ± 33.2	120.4 ± 54.7	**0.001**
Implant extraction surgery	50	18	**0.0001**
Implant migration	3	0	0.095
Implant failure	0	0	1.0
Intra-articular implant migration	0	0	1.0
Clinically significant loss of correction	0	0	1.0
Non-unions	0	0	1.0

## Data Availability

The data presented in this study are available on request from the corresponding author. The data are not publicly available due to patient privacy.
